# Action of dithiocarbimates salts on the honey bee and its pathogen *Nosema ceranae*

**DOI:** 10.1186/s13568-024-01734-z

**Published:** 2024-07-18

**Authors:** André Henrique de Oliveira, Mayura Marques Magalhães Rubinger, Anderson da Silva Rabello, Nathália Matias Albuini-Oliveira, Antonio Eustáquio Carneiro Vidigal, Marcelo Ribeiro Leite de Oliveira, Eder do Couto Tavares, José Eduardo Serrão

**Affiliations:** 1 Departamento de Biologia Geral, Universidade Federal de Viçosa, Viçosa, 36570-977 Brazil; 2 Departamento de Química, Universidade Federal de Viçosa, Viçosa, 36570-977 Brazil; 3 Instituto de Física e Química, Universidade Federal de Itajubá, Itajubá, 37500-906 Brazil

**Keywords:** *Apis mellifera*, Fungicide, Microsporidia, Pathogens

## Abstract

*Apis mellifera*, crucial pollinators for both native and cultivated plants, also yield various products such as honey, wax, royal jelly, and propolis, extensively utilized in the food, pharmaceuticals, and cosmetics industries. *Nosema ceranae*, a prevalent microsporidian worldwide, stands as a significant pathogen for *A. mellifera*, showing resistance to conventional antibiotics. Consequently, the exploration of novel compounds for *N. ceranae* control becomes imperative. Dithiocarbimate derivatives emerge as promising antifungal candidates under evaluation for combating various pathogens, particularly those affecting plants. This study assessed the toxicity profile of six dithiocarbimate derivatives on *A. mellifera* worker survival and *N. ceranae* pathogen. Among these, four compounds exhibited minimal bee mortality and proceeded to further evaluation against *N. ceranae*. *In vitro* assays demonstrated their inhibitory effects on spore germination. Remarkably, the most potent compound suppressed *N. ceranae* spores by 62% at a concentration of 20 µmol L^−1^*in vivo*. Thus, these dithiocarbimate derivatives represent promising new antifungal agents for combatting nosemosis in honey bee populations.

## Introduction

Bees serve as indispensable pollinators in terrestrial ecosystems, offering significant economic benefits to industries and agriculture, with their service valued between US$ 235–577 billion annually (Potts et al. 2016). Despite being crucial agricultural pollinators globally, *Apis mellifera* and other bee species have faced a substantial decline in populations worldwide (Castilhos et al. 2019; Zattara and Aizen 2021).

The decline in bee populations has been attributed to a multitude of stressors, including environmental pollution, habitat loss, and fragmentation, monoculture farming, climate change, improper management practices, increased pesticide use, as well as parasites and pathogens (Grab et al. 2019; Guimarães-Cestaro et al. 2020; Grant et al. 2021). Notably, among the pathogens, Microsporidia *Nosema* spp. are a threat to *A. mellifera*, resulting in mortality and reduced honey production (Burnham 2019; Goblirsch 2018; Epilobee et al. 2016).

Two species of Microsporidia infect *A. mellifera*: *Nosema apis*, first reported in 1909 (Zander 1909), and *Nosema ceranae*, originally found in *Apis ceranae* (Fries et al. 1996). Currently, *N. ceranae* is the most widespread species affecting honey bee populations globally (Goulson et al. 2015; Goblirsch 2018).

*N. ceranae* alters carbohydrate metabolism in infected bees, ensuring nutrient availability for its benefit (Dussaubat et al. 2012). Its presence can lead to colony collapse if it exceeds a critical threshold (Higes et al. 2008; Goblirsch et al. 2013).

Nosemosis has been identified as the primary cause of mortality in 54% of *A. mellifera* colonies in the USA (Seitz et al. 2016), and it has caused significant colony losses in Spain (Higes et al. 2008) and Bulgaria (Parvanov et al. 2014). In Brazil, *N. ceranae* is the sole species infecting *A. mellifera* (Teixeira et al. 2013; Guimarães-Cestaro et al. 2016a; Lage et al. 2022), suggesting its successful adaptation to the tropical climate (Martín-Hernández et al. 2009).

Bees become infected with *Nosema* spp. by ingesting spores from contaminated food and water, often while cleaning contaminated combs (Higes et al. 2010) or during trophallaxis (Smith 2012). This pathogen induces reduced longevity in adult bees, behavioral alterations, and dysentery, ultimately resulting in individual bee mortality and potential colony collapse (Anderson and Giacon 1992; Kralj and Fuchs 2010).

The treatment of nosemosis in bees has traditionally relied on the antibiotic fumagillin (Williams et al. 2008; Higes et al. 2011). However, its efficacy has waned, particularly against *N. ceranae* (Huang et al. 2013; Burnham 2019). Moreover, fumagillin’s usage has been banned in the European Union, Chile, Australia, and other regions due to concerns over chromosomal changes and carcinogenicity in humans (European Commission 2009; Botías 2012; van den Heever et al. 2014; Burnham 2019). Consequently, with no alternative antibiotics available for treating nosemosis, the pursuit of new bioactive compounds capable of controlling *N. ceranae* in *A. mellifera* becomes imperative.

Dithiocarbamates have long been utilized as broad-spectrum agricultural fungicides (Gullino et al. 2010; Ajiboye et al. 2022). Among them, mancozeb stands out as a widely used fungicide, comprising a zinc and manganese complex with the ethylene bis(dithiocarbamate) ligand, employed to combat various fungal diseases across field crops, fruits, vegetables, and ornamentals (Campanale et al. 2023). However, a significant toxicological concern arises from its environmental decomposition, which releases the thyrotropic agent ethylene thiourea (van Wendel de Joode et al. 2014; Campanale et al. 2023). Furthermore, the literature highlights the potential adverse effects of dithiocarbamates on the survival and flight capacity of *A. mellifera* and other bee species (Porrini et al. 2003; Gomes et al. 2023).

We have turned our attention to a less-explored group of compounds: dithiocarbimates derived from sulfonamides. Zinc complexes with these ligands, as well as organic allyldithiocarbimates, have shown promise in combating various plant diseases (Alves et al. 2009; Amim et al. 2011; Oliveira et al. 2015; Tavares et al. 2016; Vidigal et al. 2020; Albuini-Oliveira et al. 2020). Specifically, zinc-*N*-R-sulfonyldithiocarbimates (where R represents alkyl and aryl groups) have demonstrated efficient control of coffee leaf rust disease caused by the fungus *Hemileia vastatrix* at remarkably low doses *in vivo* (Rabello et al. 2022). Since certain dithiocarbimate derivatives used in this context did not increase the mortality of pollinators like *A. mellifera* (Rabello et al. 2022), we deemed it worthwhile to investigate their efficacy against *N. ceranae* and their potential application in controlling honey bee nosemosis.

**Fig. 1:**
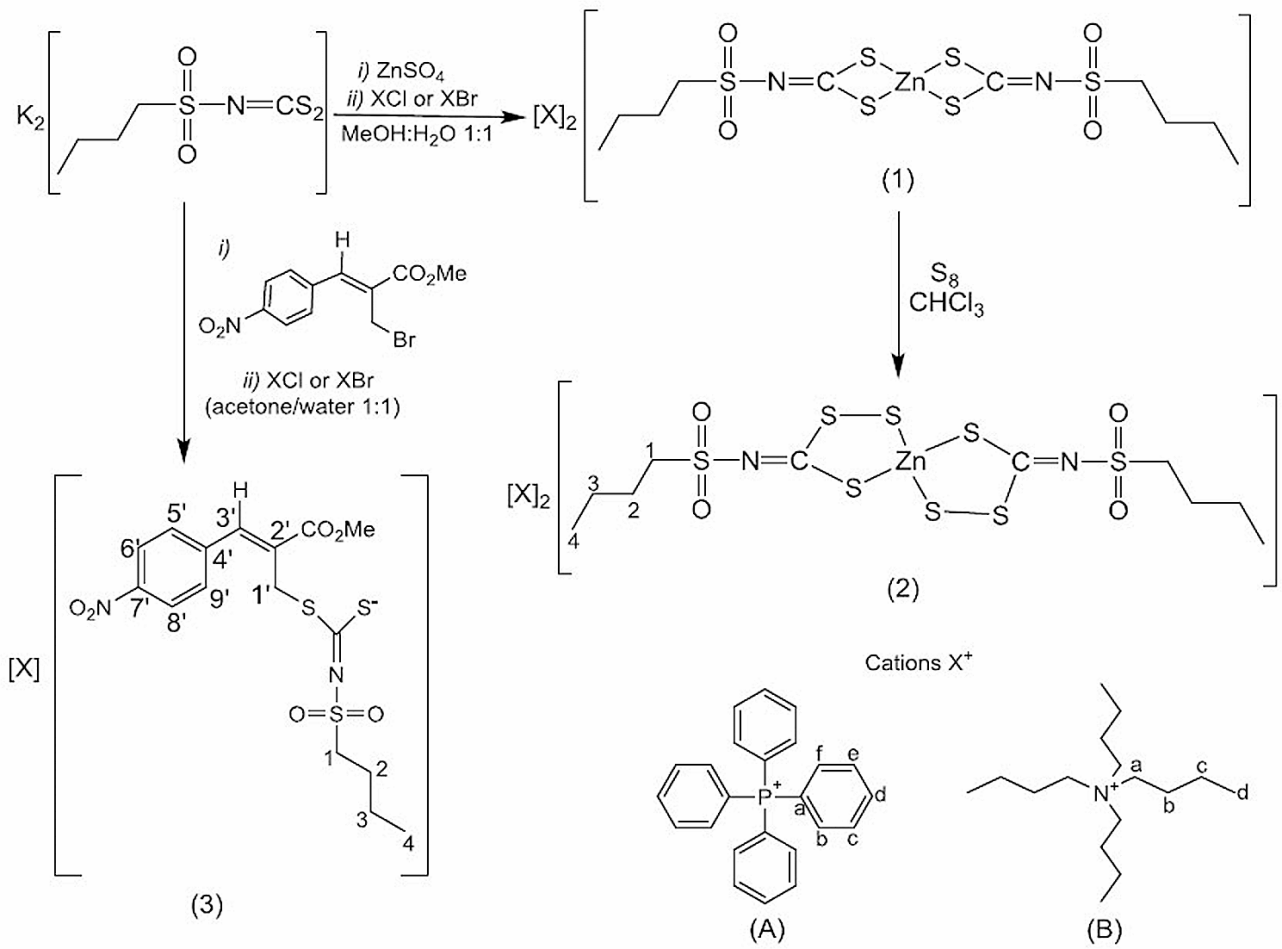
Syntheses of zinc-dithiocarbimate (1), zinc-trithiocarbimate (2), and allyldithiocarbimate (3) salts of tetraphenylphosphonium (**A**) and tetrabutylammonium (**B**), and the codes used for the NMR attributions

In contrast to dithiocarbamates, which are monoanions (R_2_N−CS_2_^-1^) and create neutral complexes with zinc(II), dithiocarbimates are dianions (RN = CS_2_^-2^). Consequently, their zinc(II) complexes (structural formula 1; Fig. 1) can be obtained as salts with various cations. This phenomenon is also observed with allyldithiocarbimates (structural formula 3; Fig. 1).

It has been previously shown that the antifungal efficacy of dithiocarbimate derivatives can be influenced by the type of cation utilized (Oliveira et al. 2015; Rabello et al. 2022), and it also relies on the nature and length of the R group (Vidigal et al. 2020; Rabello et al. 2022). For the current investigation, the butyl chain on the dithiocarbimate group (Fig. 1) and the tetraphenylphosphonium and tetrabutylammonium cations A and B (Fig. 1) were chosen based on their documented positive effects on the antifungal activity of dithiocarbimate derivatives (Oliveira et al. 2015; Albuini-Oliveira et al. 2020; Vidigal et al. 2020; Rabello et al. 2022). Additionally, the nitro-group on the aromatic ring has been shown to enhance the antifungal potency of allyldithiocarbimates against phytopathogenic fungi (Albuini-Oliveira et al. 2020), leading to its incorporation into the selected structure 3 (Fig. 1).

Zinc-dithiocarbimates undergo a reaction with sulfur, resulting in the formation of trithiocarbimates (Castro et al. 2017; Tavares et al. 2012; Oliveira et al. 2007). Given the absence of biological investigations concerning trithiocarbimates, we also explore these sulfur-rich derivatives as potential antifungals against *N. ceranae* (represented by salts 2A and 2B in Fig. 1).

The objective was to assess the toxicity of six dithiocarbimate-derived compounds on *A. mellifera* workers, alongside their fungicidal efficacy against the microsporidian pathogen *N. ceranae*, both *in vitro* and *in vivo*. Among these compounds, three are novel and underwent comprehensive characterization through spectroscopic techniques.

## Materials and methods

### Equipments

Melting points were determined with the MQAPF-302 equipment (Microquímica, Palhoça, Brazil) and are reported without correction. Elemental analyses for C, H, and N were performed using a Leco TruSpec Micro analyzer (St. Joseph, USA). The infrared (IR) spectra were recorded on a Varian 660-IR IR (Palo Alto, USA) equipped with GladiATR (Attenuated Total Reflection) scanning from 4000 to 400 cm^−1^. The nuclear magnetic resonance (NMR) spectra were obtained at 300 K using a Varian 300 MHz (Palo Alto, USA) or a Brucker Advance DRX 400 MHz (Billerica, USA) from solutions in deuterated chloroform (CDCl_3_, Sigma-Aldrich, St. Louis, USA), with tetramethyl silane (Sigma-Aldrich, St. Louis, USA) as internal standard. The exact mass of the allylditiocarbimate was determined by high-resolution electrospray ionization mass spectrometry (HRMS-ESI) in acetonitrile solutions by direct infusion method using a Bruker Daltonics MicroTOF-QII-ESI-QQ-TOF mass spectrometer (Billerica, USA).

### Reagents and synthetic precursors

The butanesulfonamide was prepared from butanesulfonyl chloride (Sigma, St. Louis, USA), in reaction with concentrated ammonia solution (Vetec, Duque de Caxias, Brazil) under reflux. The potassium *N*-butylsulfonyldithiocarbimate was prepared from the butanesulfonamide in reaction with carbon disulfide (Vetec, Duque de Caxias, Brazil) and two equivalents of potassium hydroxide (Vetec, Duque de Caxias, Brazil) in dimethylformamide (Vetec, Duque de Caxias, Brazil), and the spectroscopic data were by the literature (Cunha et al. 2010). A Morita-Baylis-Hillman adduct was prepared by the reaction of methyl acrylate (Sigma, St. Louis, USA) with 4-nitrobenzaldehyde (Sigma, St. Louis, USA), catalyzed by trimethylamine (Cai et al. 2002) and was converted into the methyl (*Z*)-2-(bromomethyl)-3-(4-nitrophenyl) acrylate, after the reaction with lithium bromide (Merck, Darmstadt, Germany) and sulfuric acid (Vetec, Duque de Caxias, Brazil), and the spectroscopic data were by the literature (Ferreira et al. 2009). The solvents (A.C.S. purity) acetone, chloroform, ethyl acetate, methanol and petroleum ether were purchased from F. Maia (Mogi das Cruzes, Brazil). The salts tetraphenylphosphonium chloride and tetrabutylammonium bromide were purchased from Alfa–Aesar (Ward Hill, USA), zinc sulfate from Merck (Darmstadt, Germany), and sulfur from Vetec (Duque de Caxias, Brazil).

### Syntheses of the zinc complexes and allyldithiocarbimate salts

The salts used in the biological essays were prepared as shown in Fig. 1.

### Zinc complexes

The zinc-dithiocarbimate salts 1A and 1B were prepared by adding 1 mmol of zinc sulfate to the stirring solution of the potassium *N*-butylsulfonyldithiocarbimate (2 mmol) in 30 mL of methanol:water (1:1 by volume). After 30 min, 2 mmol of the appropriate counterion halide were added: tetraphenylphosphonium chloride (A) or tetrabutylammonium bromide (B). After stirring for one hour, the mixtures were filtered, and the white solids were washed with distilled water (30 mL) and dried in a vacuum desiccator to constant mass.

The syntheses of the zinc-trithiocarbimate salts 2A and 2B were performed by adding elemental sulfur (1 mmol) to solutions of the zinc complexes 1A or 1B (4 mmol) in chloroform (20 mL). The mixtures were stirred for one hour at room temperature, filtered (no observable residue) and the solvent was evaporated under reduced pressure. The oily residues were triturated with 5–10 mL of petroleum ether until loose solids were obtained.

All the zinc complexes were characterized by their melting points, elemental analyses, infrared and NMR spectroscopies.

### Allyldithiocarbimate salts

A solution of methyl (*Z*)-2-(bromomethyl)-3-(4-nitrophenyl)acrylate (1 mmol) in acetone (2 mL) was added dropwise to a stirring solution of potassium *N*-butylsulfonyldithiocarbimate (1.2 mmol) in acetone:water (1:1 by volume, 10 mL). The mixture was stirred for 15 min (monitored by TLC) at room temperature. Then, water (10 mL) was added and the product was extracted with ethyl acetate (3 × 20 mL). The organic phase was concentrated under reduced pressure and the residue was dissolved in water (10 mL).

For the synthesis of salt 3A, 1 mmol of tetraphenylphosphonium chloride was added to this aqueous solution containing the allyldithiocarbimate anion. After stirring for 5 min at room temperature, the yellow solid thus formed was filtered, washed with distilled water, and dried under reduced pressure for one day.

For the synthesis of salt 3B, 1 mmol of tetrabutylammonium bromide was added to the aqueous solution containing the allyldithiocarbimate anion. The mixture was stirred for 10 min, forming an oily residue stuck to the flask walls. The water phase was discarded and the oily residue was washed with water (3 × 10 mL). The oil was dried under reduced pressure for one day.

The allyldithiocarbimates 3A and 3B were characterized by HRMS, infrared and NMR spectroscopies. The melting point of 3A was in accordance with the literature (Vidigal et al. 2020).

### Bees

Brood frames were collected from three *A. mellifera* colonies located in apiaries situated in Viçosa (20°45´S 42°52´W), in the state of Minas Gerais, Brazil. These frames were maintained at 32 ± 2 °C in darkness to obtain newly-emerged workers devoid of *Nosema* spp. Upon emergence (< 24 h old), the bees were transferred to 1 L plastic cages in groups of 320 individuals and provided with pollen and honey sourced from the colonies *ad libitum* for 24 h. Subsequently, the bees were moved to four cages (500 mL each) containing 80 workers per cage. They were fed *ad libitum* on a 50% aqueous sucrose solution and pollen grains.

### Toxicity to bees

The worker bees were confined in cages, each containing 10 individuals, and were provided *ad libitum* with pollen grains and 50% sucrose solutions supplemented with the salts derived from dithiocarbimates at two concentrations (20 and 100 µmol L^-1^), along with 0.1% dimethylsulfoxide (DMSO, Merck, Darmstadt, Germany) as a co-solvent to ensure homogeneity. Additionally, toxicity tests were conducted using 0.1% DMSO in 50% sucrose solution as a control, which was compared to the control group fed on only 50% sucrose solution. Worker survival was assessed at 16 h, 24 h, and 48 h post-treatment. Each test was performed in triplicate and repeated twice.

#### Isolation of *N. ceranae* spores

To obtain *N. ceranae* spores, naturally infected colonies of *A. mellifera* were utilized. Prior confirmation of infection was conducted through the examination of workers using light microscopy, as detailed in previous studies (Guimarães-Cestaro et al. 2016b). In brief, bees were dissected, and their midguts were macerated in 1 mL of distilled water per individual. The resulting macerate was then filtered through cotton wool and centrifuged at 5000 g for 5 min, repeated three times. The pellet was resuspended in distilled water and homogenized using vortexing. Subsequently, an aliquot of the suspension was analyzed using a Neubauer chamber (Assistent, Sondheim, Germany) to count and determine the concentration of spore present in the suspension (Fries et al. 2013).

#### Bee infection with *N. ceranae*

Newly-emerged bees, aged up to 24 h, were transferred to cages containing 80 workers each and starved for three hours. Subsequently, the bees were fed for 24 h with 800 µL of a 50% sucrose solution containing isolated spores of *N. ceraneae* at a concentration of 12,500 spores µL^−1^. Considering the consumption of 10 µL per bee (Williams et al. 2013), it was estimated that each bee received approximately 125,000 spores within 24 h. Following exposure, all bees, including control, received a spore-free 50% sucrose solution until the conclusion of the experiment.

#### Antifungal activity *in vitro*

*N. ceranae* spores, obtained as described, were exposed in five replicates to each dithiocarbimate derivative with low toxicity for bees, as estimated previously, for 1 h at room temperature. The spore suspensions were then incubated with 10 µg mL^−1^ of 4′,6-diamidino-2-phenylindole (DAPI, Merk, Darmstadt, Germany) for 30 min. DAPI is a fluorescent dye that penetrates only non-viable spores. After incubation, the spores were washed twice in distilled water, centrifuged at 5000 *g *for 5 min, and resuspended for counting in a Neubauer chamber using a fluorescence microscope. For each evaluation, the spores were first counted under fluorescence and then in a bright field microscope. Spores stained with DAPI were considered non-viable (McGowan et al. 2016).

#### Antifungal activity *in vivo*

The dithiocarbimate derivatives, previously determined to have low toxicity for bees, were dissolved in 50% sucrose solution and provided ad libitum for feeding the 20 infected bees. Seven days after feeding, 10 bees were dissected, and their midguts were used to obtain and count the viable spores.

### Statistical analysis

The data on viable spores in vitro and the quantity of spores in the midgut of *A. mellifera* from the control and treated groups were submitted to one-way ANOVA. The results were compared post-hoc with the Tukey test at 5% significance, using the SISVAR software version 5.6 (Ferreira 2011).

## Results

### Syntheses

The syntheses performed as shown in Fig. 1 were caried out in good yields: 93% for 1A and 85% for 1B, 89% for 2A, 82% for 2B, 80% for 3A and 81% for 3B. The salts 1A, 1B, and 3A have been published and their spectroscopic data were in accordance with the literature (Cunha et al. 2012; Rabello et al. 2022; Vidigal et al. 2020).

The compounds 2A, 2B and 3B are new substances and their characterization data are as follows:

Tetraphenylphosphonium *bis* (butylsulphonyltrithiocarbimato)zincato(II), (2A): m.p. 61.6–62.8 °C; Found (Calcd.) for C_58_H_58_N_2_O_4_P_2_S_8_Zn: C, 57.95 (56.59); H, 4.78 (4.75); N, 2.27 (2.28); IR (selected bands, ATR) ν/cm^−1^ 1382 (νCN), 1264 (ν_as_SO_2_), 1105 (ν_sym_SO_2_), 925 (νCS_3_); ^1^H NMR (300 MHz, CDCl_3_) *δ* 0.81 (t, *J* = 6 Hz, 6 H, H4), 1.24–1.38 (m, 4 H, H3), 1.70–1.82 (m, 4 H, H2), 3.11–3.21 (m, 4 H, H1), 7.59–7.68 (m, 16 H, Hb, Hf), 7.71–7.80 (m, 16 H, Hc, He), 7.81 − 7.89 (m, 8 H, Hd); ^13^C NMR (75 MHz, CDCl_3_) *δ* 13.9 (C4), 22.0 (C3), 25.7 (C2), 51.6 (C1); 117.6 (d, *J* = 90.0 Hz, Ca), 130.8 (d, *J* = 7.5 Hz, Cb e Cf), 134.6 (d, *J* = 7.5 Hz, Cc e Ce), 135.7 (d, *J* = 3.0 Hz, Cd), 208.1 (C=N).

Tetrabutylammonium *bis*(butylsulphonyltrithiocarbimato)zincato(II), (2B): m.p. 80.2–81.5 °C; Found (Calcd.) for C_42_H_90_N_4_O_4_S_8_Zn: C, 47.95 (48.64); H, 9.14 (8.75); N, 5.49 (5.40); IR (selected bands, ATR) ν/cm^−1^ 1391 (νCN), 1265 (ν_as_SO_2_), 1111 (ν_sym_SO_2_), 935 (νCS_3_); ^1^H NMR (300 MHz, CDCl_3_) *δ* 0.91 (t, *J* = 6.0 Hz, 6 H, H4 ), 0.99 (t, *J* = 6.0 Hz, 24 H, Hd), 1.40–1.47 (m, 20 H, H3, Hc), 1.63–1.80 (m, 20 H, H2, Hb), 3.22–3.70 (m, 24 H, H1, Ha); ^13^C NMR (75 MHz, CDCl_3_) *δ* 14.0 (C4, Cd), 20.0 (Cc), 22.0 (C3), 24.2 (Cb), 25.8 (C2), 52.2 (C1), 58.9 (Ca), 208.4 (C=N).

Tetrabutylammonium (*Z*)*-*2-(methoxycarbonyl)-3‑(4‑nitrophenyl)allyl-(*N*-butylsulfonyl)dithiocarbimate (3B): HRMS-ESI m/z, calcd. for C_16_H_19_N_2_O_6_S_3_^−^: 431.0411, found: 431.0355; IR (selected bands, ATR) ν/cm^−1^ 1714 (νC= O), 1520 (ν_as_NO_2_), 1382 (νCN), 1342 (ν_sym_NO_2_), 1261 (ν_as_ SO_2_), 1149 (νSO_2_), 937 (ν_as_ CS_2_); ^1^H NMR (400 MHz, CDCl_3_) *δ* 0.91 (t, *J* = 7.4 Hz, 3H, H4),1.00 (t, *J* = 7.3 Hz, 12H, Hd), 1.40–1.50 (m, 10H, H3, Hc), 1.62–1.70 (m, 8H, Hb), 1.77–1.81 (m, 2H, H2), 3.25–3.29 (m, 8H, Ha), 3.50 (t, *J* = 8.0 Hz, 2H, H1), 3.83 (s, 3H, OCH_3_), 4.21 (s, 2H, H1’), 7.21 (s, 1H, H3’), 7.77 (d, *J* = 8.8 Hz, 2 H, H5’, H9’), 8.25 (d, *J* = 8.7 Hz, 2 H, H6’, H8’); ^13^C NMR (100 MHz, CDCl_3_) *δ* 13.7 (Cd, C4), 19.8 (Cc), 21.9 (C3), 24.0 (Cb), 25.7 (C2), 33.1 (C1’), 51.2 (C1), 52.4 (OCH_3_), 58.9 (Ca), 123.8 (C6’, C8’), 130.8 (C3’), 131.2 (C2’), 138.1 (C5’, C9’), 141.4 (C4’), 147.5 (C7’), 167.4 (C = O), 199.7 (C=N).

### Dithiocarbimate salts and toxicity to bees and *N. ceranae*

Exposure of *A. mellifera* workers to dithiocarbimate-derived salts aimed to assess potential toxic effects. The toxicity results varied among the compounds (F = 117.81; *p* < 0.01; Table 1). Treatments containing salts 1B (20 µmol L^−1^), 2B (20 µmol L^−1^), 3A (20 µmol L^−1^), and 3B (20 µmol L^−1^) exhibited mortality rates similar to the control group. The predominance of safer salts featuring the tetrabutylammonium cation (B) suggests that the tetraphenylphosphonium cation (A) might contribute to the observed toxicity in other treatments (Table 1). Subsequent tests focused solely on compounds resulting in up to 5% mortality among *A. mellifera* worker bees.

**Table 1:** Percentage mortality (mean ± sd) of *Apis mellufera* workers fed on dithiocarbimate compounds in two concentrations

Compound (µmol L^−1^)	Mortality (%)
1A (20)	6.6 ± 1.5^a, b^
1A (100)	63.3 ± 4.0^a^
1B (20)	5.0 ± 2.4^b^
1B (100)	23.0 ± 5.3^a^
2A (20)	26.6 ± 3.2^a^
2A (100)	58.3 ± 7.5^a^
2B (20)	5.0 ± 2.3^b^
2B (100)	26.6 ± 5.1^a^
3A (20)	0^b^
3A (100)	46.6 ± 8.1^a^
3B (20)	0^b^
3B (100)	11.6 ± 4.0^a^
DMSO 0.1%	0^b^
Control	0^b^

Different letters in the column indicate significant difference among compounds and the controls based on Tukey test at 5% of significance level

**Fig. 2:**
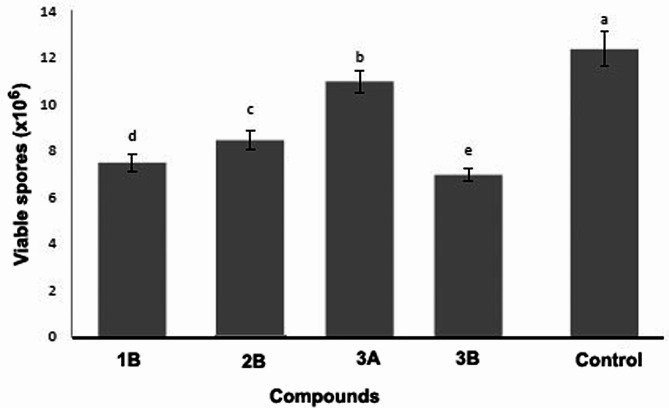
Number (mean ± sd) of viable *Nosema ceranae* spores after exposure in vitro to compounds derived from dithiocarbimates and control group. Different letters above bars indicate significant difference among compounds based on Tukey test at 5% of significance level obtained from five independent biological replications

In vitro exposure of *N. ceranae* spores to the four selected compounds resulted in a decrease in viability rates, with counts dropping from 12.37 ± 0.74 × 10^6^ viable spores in the control to 11.10 ± 0.47 × 10^6^ with 3A, 8.39 ± 0.40 × 10^6^ with 1B, 6.96 ± 0.28 × 10^6^ with 2B, and 7.56 ± 0.38 × 10^6^ with 3B (F = 419.950; *p* < 0.001; Fig. 2).

The in vitro antifungal activity of salts 1B, 2B, 3B, and 3A demonstrated reductions in *N. ceranae* spore viability ranging from 10 to 44%, with the most significant improvements observed in treatments containing salts with the tetrabutylammonium cation (B). Additionally, variations in spore viability were evident based on the type of dithiocarbimate derivative used, as treatments 1B, 2B, and 3B exhibited distinct reductions in *N. ceranae* spore viability by 32%, 44%, and 39%, respectively.

**Fig. 3:**
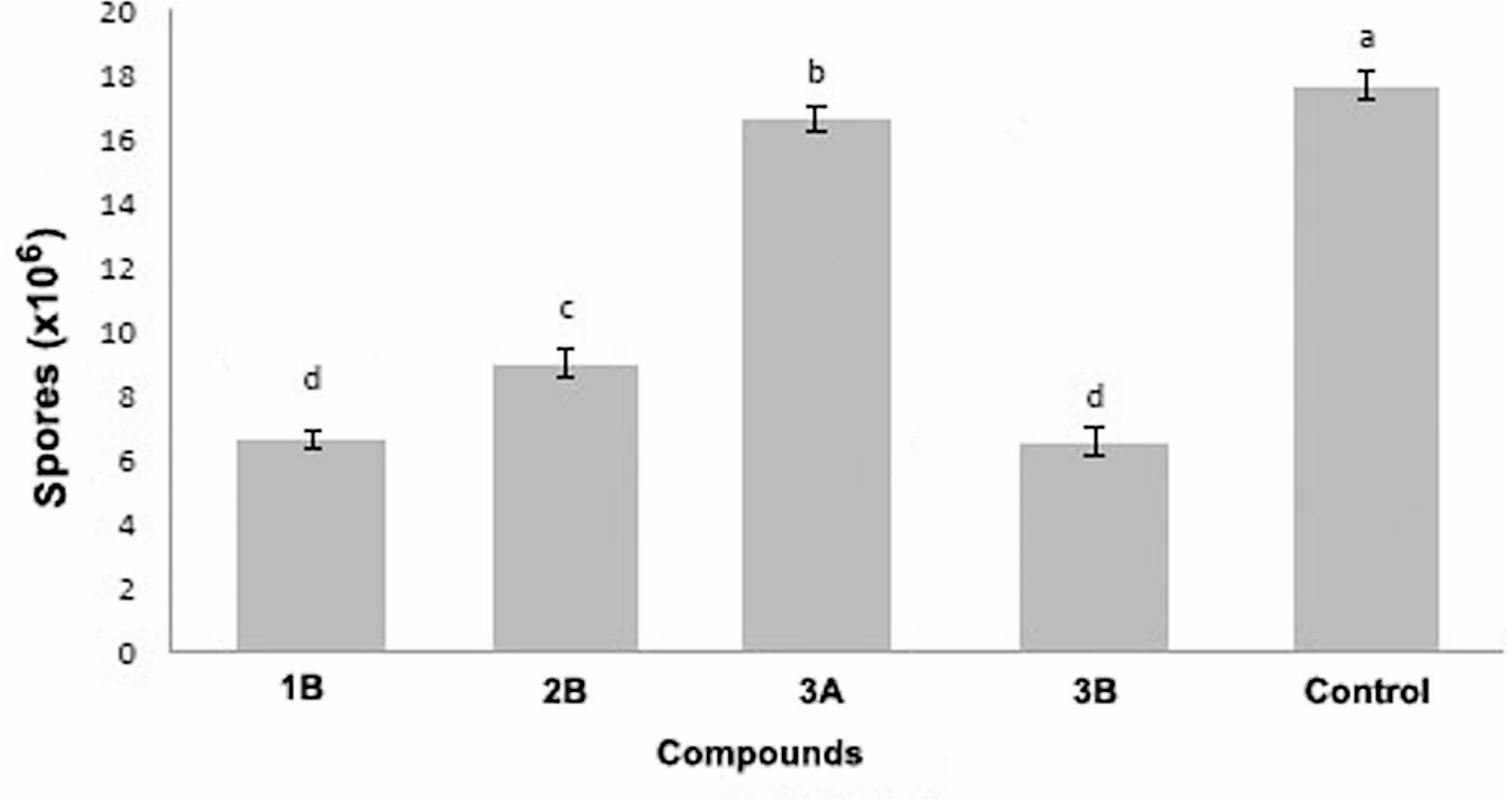
Number (mean ± sd) of *Nosema ceranae* spores in the midgut of *Apis mellifera* eight days post-inoculation, after treatments in vivo with compounds derived from dithiocarbimate. Different letters above bars indicate significant difference among compounds based on Tukey test at 5% of significance level obtained from 10 independent biological replications

The in vivo antifungal activity was assessed by quantifying the spore quantities in the midgut of infected honey bees fed on the four compounds. Results revealed significant differences among treatments on the eighth-day post-inoculation (F = 30.688; *p* < 0.001), ranging from 17.66 ± 0.46 × 10^6^ spores per midgut in the control to 9.01 ± 0.46 × 10^6^ with 2B, 6.65 ± 0.30 × 10^6^ with 1B, 6.56 ± 0.42 × 10^6^ with 3B, and 16.63 ± 0.39 × 10^6^ with 3A (Fig. 3).

The in vivo test confirmed the efficacy of salts 1B, 2B, and 3B, resulting in a reduction ranging from 49 to 63% in the concentration of *N. ceranae* spores in the midgut of *A. mellifera* workers. Particularly noteworthy were the findings of treatments with salts 1B (62% inhibition) and 3B (63% inhibition), demonstrating promising antifungal activity against this Microsporidia.

## Discussion

The zinc-dithiocarbimate anions (1) form stable salts with the cations A and B (Fig. 1) (Cunha et al. 2012; Rabello et al. 2022). While these substances are white solids, the zinc-trithiocarbimate salts 2A and 2B are yellow. Although analogous zinc complexes have been prepared with other trithiocarbimates (Tavares et al. 2012; Castro et al. 2017), the salts 2A and 2B are new substances. Their formulae were supported by elemental analyses, which also confirmed their purity.

In addition to the color difference, the zinc-trithiocarbimate salts 2A and 2B can be distinguished by their lower melting points compared to the parental complexes 1A and 1B. For instance, while the salt 2B melts at 80.2–81.5 °C, the corresponding zinc-dithiocarbimate salt 1B exhibits an onset melting at 122 °C (Rabello et al. 2022).

The integration curves observed in the ^1^H NMR spectra of salts 2A and 2B were consistent with a 2:1 ratio between the tetraphenylphosphonium or tetrabutylammonium cations and the zinc-trithiocarbimate anion. The signals corresponding to the trithiocarbimate moiety in both the ^1^H and ^13^C NMR spectra of the complexes exhibited chemical shifts similar to those observed in the parental zinc-dithiocarbimates (Rabello et al. 2022). In the ^13^C NMR spectra of 2A and 2B, most signals showed slight upfield shifts compared to the spectra of the parent complexes. For instance, signals attributed to the butyl group in complex 1B, observed around 13, 21, 25, and 51 ppm (Rabello et al. 2022), shifted to approximately 14, 22, 26, and 52 ppm in the spectrum of salt 2B.

The infrared data obtained for the salts 2A and 2B are also similar to those observed for the parental compounds 1A and 1B (Cunha et al. 2012; Rabello et al. 2022), though slight shifts are observed for each band. For example, while the C= N stretching band appears at 1385 cm^−1^ in the spectrum of 1B (Rabello et al. 2022) the value for compound 2B is 1391 cm^−1^.

The allyldithiocarbimate anion of 3A and 3B has been previously prepared and isolated as a tetraphenylphosphonium salt, as 3A (Vidigal et al. 2020). The spectroscopic data of the new salt of tetrabutylammonium (salt 3B) are very similar to those of 3A, concerning the signals and bands due to the allyldithiocarbimate anion. Any disparities noted between the spectra of 3A and 3B solely due to the distinct cations A and B. Nevertheless, the two substances are easily recognizable by their melting points. While the salt 3A melts at 120.7−122.6 ^o^C, 3B is a viscous oil at room temperature.

The molecular formula of salt 3B was confirmed via HRMS, which exhibited the expected peak corresponding to the tetrabutylammonium ion in the positive mode, alongside the molecular ion peak of the allyldithiocarbimate in the negative mode. Additionally, the integration curves observed in the ^1^H NMR spectrum of salt 3B were consistent with a 1:1 ratio between the allyldithiocarbimate anion and the tetrabutylammonium cation.

It is worth noting the chemical shift of the C = N signal, which transitions from 223.6 ppm in the spectrum of potassium *N*-butylsulfonildithiocarbimate (Cunha et al. 2010) to approximately 208 ppm in the spectra of zinc complexes 1A, 1B, 2A, and 2B, and to ca. 200 ppm in the spectra of allyldithiocarbimates 3A and 3B. This shift suggests an increased shielding effect on this carbon atom (Rabello et al. 2022; Vidigal et al. 2020). This observation aligns with the IR data, which indicates a greater double bond character of the C = N bond in the allyldithiocarbimates and the complexes compared to the free dithiocarbimate ligand. The stretching of the C = N bond gives rise to a band at 1283 cm^−1^ in the IR spectrum of potassium *N*-butyldithiocarbimate (Cunha et al. 2010), with corresponding bands observed at approximately 1390 cm^−1^ for derivatives 1A, 1B, 2A, 2B, 3A, and 3B (Rabello et al. 2022; Vidigal et al. 2020).

Currently, the antibiotic fumigallin is the main chemical used to control nosemosis in vivo, but it has been reported to have low efficacy against *N. ceranae* in some apiaries (Huang et al. 2013; Burnham 2019). So, new compounds have been evaluated to control this Microsporidia, including the natural compounds resveratrol and thymol (Maistrello et al. 2008; Glavinic et al. 2022), formic acid (Underwood and Currie 2009), the phyto-pharmacological preparation Novezit (Higes et al. 2014), several plant extracts (Chaimanee et al. 2021), and the synthetic compounds fenbendazole and ornidazole (Bahreini et al. 2022), which have been showed some potential to control but almost always in high doses and with lower efficacy than fumigallin. Whereas the compound here evaluated showed a mortality to *N. ceranae* of ca. 40% in vitro and 60% in vivo.

The superior reduction of *N. ceranae* spores observed in vivo compared to the in vitro test suggests the involvement of at least two modes of action into the insect body treated with the dithiocarbimate derivatives. While the precise mode of action of these compounds on Microsporidia remains unexplored, similar compounds like dithiocarbamates have been shown to act through oxidative mechanisms on the amino acid cysteine, abundant in proteins from the polar tube of *Nosema bombycis* infecting silkworms (Dias et al. 2010; Lv et al. 2020). However, further studies are necessary to elucidate the specific modes of action of the compounds here evaluated.

This study sheds light on potential applications and ecological responses of salts derived from dithiocarbimates, revealing that their toxicity towards *A. mellifera* worker bees can vary depending on the specific derivative. Notably, the tetrabutylammonium cation (B) emerged as the preferable option over the tetraphenylphosphonium cation (A), particularly in terms of reduced toxicity to bees. Overall, the four tetrabutylammonium salts investigated in this study exhibit potential in controlling *N. ceranae* spores, demonstrated by the decrease of spore viability in vitro and spore quantities in the midgut of infected *A. mellifera* workers.

## Data Availability

All data generated in this study are described in the published article. Raw data may be provided upon reasonable request from the corresponding authors.
